# Acute renal infarction, transient ischemic attack, and biventricular thrombi secondary to substance use disorder: A case report

**DOI:** 10.1002/ccr3.2101

**Published:** 2019-03-19

**Authors:** Azadeh Ghassemi, Juma Bharadia, Antonio Liu

**Affiliations:** ^1^ Department of Neurology California Hospital Medical Center Los Angeles California; ^2^ Ross University School of Medicine Miramar Florida; ^3^ Department of Cardiology California Hospital Medical Center Los Angeles California

**Keywords:** biventricular thrombi, prothrombic events, renal infarct, substance use disorder, transient ischemic attack

## Abstract

Cocaine is the second most used illicit drug; cocaine induces platelet activation and formation of thrombus. Thrombotic effects of cocaine can lead to vascular injuries, cerebrovascular accident and myocardial infarct. Less common, cocaine use disorder leads to thrombi formation in both ventricles and renal artery infarct as seen in our patient.

## 1 INTRODUCTION

Isolated left or right ventricle thrombus has been well documented; however, biventricular thrombi are rare.^1^ Cases of biventricular thrombi are often reported in patients with prothrombic conditions such as, antiphospholipid antibody syndrome (APS) and heparin‐induced thrombocytopenia, and in patients with Libman‐Sacks endocarditis.^2^, ^3^, ^4^

This paper reports a case of biventricular cavitary thrombi that presented with a transient ischemic attack and left renal artery infarct in a young Hispanic patient with history of substance use disorder.

## 2 CASE REPORT

A 26‐year‐old Hispanic male presented to the emergency department of our hospital with acute onset of numbness and weakness in his left arm. Shortly after his arrival, he began to experience a pressure like pain in his left upper abdominal quadrant (LUQ) which was nonradiating and moderate in severity. His neurological symptoms resolved within one hour of presentation; however, his LUQ abdominal pain worsened and was associated with repeated vomiting episodes. The patient had history of smoking (2‐3 cigarettes per day), drinking hard liquor daily, and taking illicit drugs (cocaine and marijuana).

The initial evaluation included a CT of the head with and without contrast, a CTA of the cervical carotids and intracranial vessels, and MRI of the brain without contrast; all these imaging tests were negative for any acute intracerebral hemorrhage, infarct, or other abnormalities (aneurysm or obstruction) in the vessels of the head or the neck.

Given the nature of the abdominal pain, both noncontrast‐ and contrast‐enhanced helical abdominal CT images were obtained. CTA of renal arteries revealed a left renal infarct with a filling defect.

**Figure 1:**
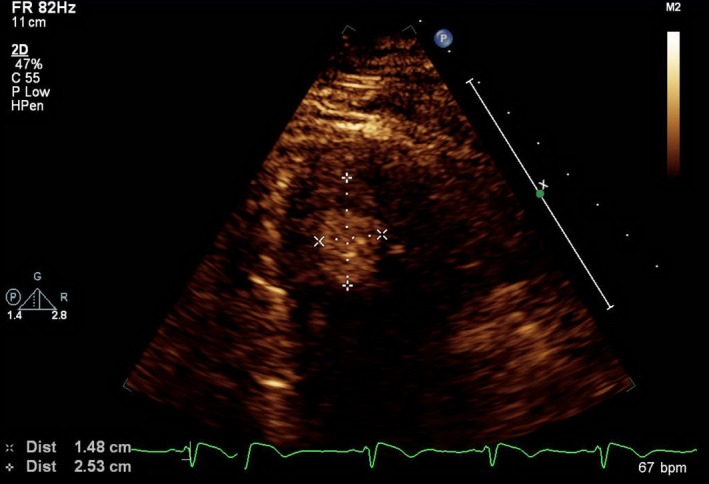
Biventricular apical thrombi in RV

**Figure 2:**
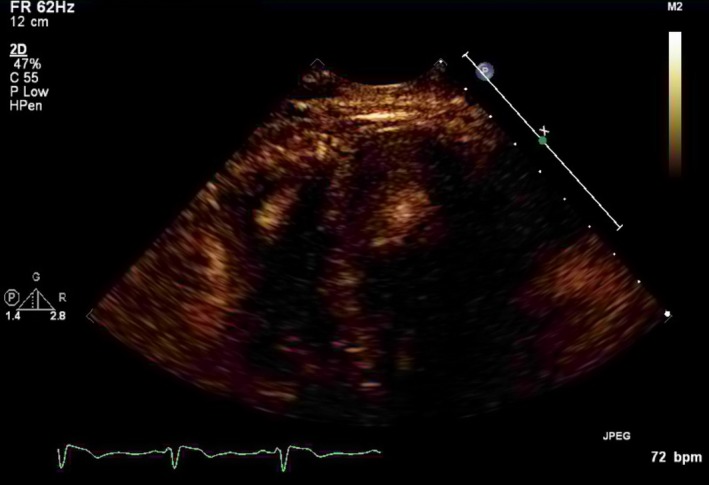
LV thrombus

Transthoracic echocardiogram showed mild to moderate dilated left ventricle with ejection fraction of 55%‐60%. The right ventricle was also mildly dilated with grossly normal systolic function. Two oval, mobile, and pedunculated hyperechoic masses, a 2.4 × 1.5 cm in LV and a 2.3 × 1.1 cm in RV, were identified (Figures 1 and 2).

A thrombus is identified as a discrete echo‐dense mass with well‐defined borders which is seen throughout a cardiac cycle.^5^

Coagulation studies including screening, mixing, and confirmatory studies were performed, and the results revealed the presence of lupus anticoagulant (LA) (Table 1).

**Table 1:** Coagulation studies of our patient

Parameters	Results
PT	22.3
PTT	83 s
INR	1
LA‐PTT	145.4 s
dRVVT confirm ratio	1.2
dRVVT screen	52.1 s
Antithrombin antigen	79% (low)
Fibrinogen level	410.9 mg/dL (High)
Hexagonal confirmatory	<8.0 s
Protein C functional	71%
Protein S functional	91.6%
Anticardiolipin IgG	<1.6 GPL (negative)
Anticardiolipin IgM	0.8 MPL (negative)

Patient was treated with proper anticoagulants; his symptoms were resolved; unfortunately, patient was lost to follow up.

## 3 DISCUSSION

Incidence rate of intracardiac thrombi (ie, biventricular thrombi) is rare; however, when present, it can lead to multiple arterial emboli.^2^, ^3^, ^4^ As seen in this patient, cardiac thrombi led to left renal infarct and transient ischemic attack (TIA).

Our patient presented initially with left‐sided hemiparesis, due to his clinical presentation with his negative head CT and MRI, a diagnosis of TIA was concluded. His left upper quadrant abdominal pain which was associated with vomiting was nonspecific; therefore, renal artery imaging was necessary to rule in or rule out renal artery defect in time; CTA of renal vasculature revealed a left renal artery defect. Given his young age, history of smoking, substance use disorder, and absence of any chronic diseases, a blood clotting disorder was suspected. Coagulation studies including screening, mixing, and confirmatory studies were performed, and the results revealed the presence of LA.

Our patient was not previously diagnosed with any blood clotting disorder (hypercoagulable states). One of the leading causes of acquired thrombophilia is APS.^6^ APS can be isolated (primary APS) or related to systemic lupus erythematous (SLE) (secondary APS).^7^ In APS, phospholipid antibodies attach to a negatively charged phospholipid surface which induces platelet activation that interferes with the function of coagulation inhibitor proteins such as, protein C and S.^8^, ^9^ Thromboembolic events in APS occur in both arteries and veins with higher incidence rate of venous thromboembolism.^6^

Patients with APS often present with TIA or CVA secondary to arterial thrombi and cerebral ischemia. Renal infarct is another manifestation of APS. There is also an increased risk of intracardiac thrombosis in APS.

Renal infarct is the result of occlusion of arterial blood supply to one or both kidneys often secondary to thromboembolism.^7^ The clinical presentation of abdominal pain can be nonspecific; therefore, renal artery imaging is necessary to make the diagnose of renal artery defect in time and to prevent long‐term complications due to delayed diagnosis.^10^

Therefore, vascular events are common in patients who are diagnosed with APS. However, in the case of our patient, the vascular events such as, TIA and renal filing defect with embolus, were the first manifestation of a hypercoagulable state.

The diagnosis of APS is often made based on the application of the Sapporo criteria (Table 2).

**Table 2:** Sapporo criteria

Clinical criteria:
Vascular thrombosis
Pregnancy morbidity
Laboratory criteria^a^:
Anticardiolipin antibodies (IgG/IgM)
Anti‐β‐glycoprotein I antibodies (IgG/IgM)
Lupus anticoagulant

a >2 occasions, 12 wk apart.

This patient meets the clinical part of the Sapporo criteria (vascular thrombosis). The laboratory criteria are partially fulfilled since positive LA was not repeated in 12 weeks as the patient was lost to follow up.

This patient does not meet the full criteria for APS diagnosis; however, there is another important risk factor that contributes to his presentation. That risk factor is his history of substance use disorder, cocaine, and marijuana. Cocaine is the second most used illicit drug, and cocaine‐related health problems have inundated emergency departments across the United States.^11^ As a sympathomimetic agent, cocaine works by inhibiting the reuptake of norepinephrine and dopamine in the postsynaptic terminals which results in overstimulation of alpha‐ and beta‐adrenergic receptors.^12^ Hypertensive crises and vascular complications with either hemorrhagic or thrombotic in origin are well‐known complications of cocaine use; in addition to vascular changes, cocaine also has prothrombic effects.^11^ Cocaine use can lead to cocaine‐induced myocardia infarct, transient ischemic accidents, and kidney damage (other than renal infarct) which are well known in the literature; however, cocaine‐induced renal infarct is not as common.^13^ The pathophysiology of cocaine‐induced vascular injury is multifactorial; endothelium dysfunction induced by cocaine results in increased platelet activation and aggregation.^13^ Cocaine also enhances plasminogen activator inhibitor activities (PAI‐I).^14^ Enhanced platelet activities and PAI‐I lead to a transient hypercoagulopathy state with potential for life‐threatening coronary arterial thrombosis and spontaneous embolization.^11^, ^15^ Furthermore, concurrent cigarette smoking with the use of cocaine can potentiate the prothrombic events.^11^ Prothrombic effects of cocaine may have contributed to the formation of thrombi in both ventricles in this patient. Cocaine‐induced coronary vessels thrombotic events have been documented; however, the case of biventricular thrombi secondary to cocaine use is rare.^16^

## 4 CONCLUSION

In summary, we demonstrated a young patient with transient ischemic attack, left renal artery infarct, and biventricular thrombi. A high index of suspicion was held for blood clotting disorder due to his young age and his history of substance use disorder. However, coagulation studies fail to support the diagnosis of antiphospholipid syndrome. His multiple symptoms from left‐sided hemiparesis to abdominal pain with vomiting led to extensive imaging studies that revealed left renal artery infarct and biventricular thrombi. Due to his history of smoking cocaine and marijuana along with cigarette smoking, we believe that his symptoms were secondary to his substance use disorder.

## CONFLICT OF INTEREST

The authors declared no potential conflicts of interest with respect to the research, authorship, and/or publication of this article.

## AUTHOR CONTRIBUTION

AL: is a practicing neurohospitalist at CHMC where the present case report took place, was this patient's neurologist at CHMC, is the primary investigator for this case report, devised and supervised this manuscript. JB: is a practicing cardiologist at CHMC, was this patient's cardiologist at CHMC, provided critical feedback and helped shape the manuscript. AG: is a 4th year medical student who attends CHMC for her clinical rotations, wrote the manuscript with support from Drs. Liu and Bharadia. All authors discussed the results and contributed to the final manuscript.
